# Evaluation of rationality of promotional drug literature using World Health Organization guidelines

**DOI:** 10.4103/0253-7613.70020

**Published:** 2010-10

**Authors:** Smita N. Mali, Sujata Dudhgaonkar, N.P. Bachewar

**Affiliations:** Department of Pharmacology, LTMC, Sion, Mumbai, India; 1 Department of Pharmacology, GMC, Nagpur, India; 2 Department of Pharmacology, SVNGMC, Yavatmal, India

**Keywords:** Brief prescription information, drug marketing, medicine promotion, promotional literature

## Abstract

**Objectives:**

The study was aimed to evaluate collected drug promotional brochures for accuracy, consistency, and validity of the information presented in it, using World Health Organization (WHO) criteria for ethical medicinal drug promotion. Drug promotional brochures were evaluated for the type of claims and pictorial content presented in it and for references cited in support of these claims.

**Material and Methods:**

This observational, cross-sectional study was conducted in the outpatient department of Government Medical College and Hospital, Nagpur, India. In addition to the fulfillment of “WHO criteria, 1988,” we examined 513 promotional brochures for the type of claims and pictorial content presented in it and references quoted in support of claims to check their retrievability, type, and authenticity.

**Results:**

None of the promotional literature fulfilled all WHO criteria. Majority (92%) brochures claimed about the efficacy of product, and a few about safety (37.8%). Out of 1003 references given in support of various claims, 84.4% were from journals and only 28.5% were validly presented researches. Brochures presenting irrelevant pictures were 41.3%, whereas brief prescription information (BPI) of the promoted drug was given only by 8.8% brochures.

**Conclusion:**

Pharmaceutical industries did not follow the WHO guidelines while promoting their products, thus aiming to satisfying their commercial motive rather than fulfill the educational aspect of promotion.

## Introduction

Pharmaceutical companies are in the business of developing and selling new drugs. These are accepted in health care system through health care professionals, and its availability is of little value unless the prescriber is aware of its existence and has scientific information to use it effectively.[1] Pharmaceutical promotion is a persuasive communication and the major marketing technique of pharmaceutical companies is “direct to physician marketing.” Physicians are contacted by medical representatives, presented with sample drugs, token gifts, reminder articles and also targeted through sponsored continued medical education, advertisements in the medical journals, etc.[2] One of the well-known promotional activities of pharmaceutical industries is to produce advertising brochures which at times are inaccurate and of poor educational value.[3–5] These promotional activities create the potential for inappropriate prescribing practices by influencing physicians’ prescribing behavior without necessarily benefiting the patients[6–8] but contributes to increased health care costs.[9]

Promotional activities by pharmaceutical companies are governed by Organization of Pharmaceutical Producers of India (OPPI), self-regulatory code of pharmaceutical marketing practices, January (2007)[10] and by National legislation.[11] Adherence to the code of conduct is a condition of membership for manufacturers’ association.[10] However, many studies have illustrated that information disseminated through drug advertisements is inconsistent with the code of ethics.[12–15] However, very few studies have been carried out in Indian setup. We decided to evaluate the rationality of the promotional drug literature as per “World Health Organization criteria for ethical medicinal drug promotion, 1988” since it is the backbone of self-regulatory code of OPPI and International Federation of Pharmaceutical Manufacturers and Associations (IFPMA) which is supposed to regulate the promotional activity of pharmaceutical industries.

## Material and Methods

This study was conducted to find out the accuracy and ethical status of promotional drug literature presented to prescribers by using “WHO criteria for ethical medicinal drug promotion, 1988”.[16] We also evaluated the drug promotional brochures for the type of claims and pictorial content presented in it and the references quoted in support of claims to check their retrievability, type, and authenticity of presentations.

This observational, cross-sectional study was conducted inthe outpatient department (OPD) of Government Medical College and tertiary care hospital at Nagpur, India, after its approval by Institutional Ethics Committee. Approximately thousand leave behind brochures were collected randomly from various OPDs, namely medicine, surgery, psychiatry, obstetrics and gynecology, ophthalmology, skin, pediatrics, and orthopedics over the period from 1^st^ October 2007 to 31^st^ March 2008. Some brochures were presented along with the reprint of journal article quoted in it as a reference to the information. Collected brochures were then explored to exclude the following materials: Literature promoting medicinal devices and equipments (insulin pump, blood glucometer, etc.), orthopedic prosthesis and ayurvedic medicines, drug monographs, reminder advertisements (reminder advertisements do not present any therapeutic information and have different criteria for evaluation),[16] drugs name list, and literature promoting more than four brands.

WHO criteria for ethical medicinal drug promotion dictate that promotional literature should contain following information.[16]

1. The name(s) of the active ingredient(s) using either international nonproprietary names (INN) or the approved generic name of the drug.
2. The brand name
3. Amount of active ingredient(s) per dose
4. Other ingredients known to cause problems, i.e. adjuvant
5. Approved therapeutic uses
6. Dosage form or dosage schedule
7. Safety information including side effects and major adverse drug reactions, precautions, contraindications and warnings, and major drug interactions
8. Name and address of manufacturer or distributor
9. Reference to scientific literature as appropriate

We evaluated criteria six separately as “dosage form” and details about “regimen” to look for the completeness of therapeutic information given in the promotional brochures. In addition to this information, promotional materials made various claims about the medicinal products presented in it. Claims made in the promotional brochures were classified into following seven categories.

### (1) Efficacy

Statements about improved effectiveness of promoted drug as a disease outcome or a patient outcome solely or in comparison with other group of drug for similar outcome (e.g., antihypertensive action of calcium channel blocker and β-blocker) or another brand of the same drug (e.g., zintac or rantac for ranitidine).

### (2) Safety

Use of the word “safe” in the promotional text or the mention of reduction in adverse drug reaction and/or drug interaction and/or contraindication.

### (3) Cost

Pointing out low price of promoted drug in absolute or relative terms or any description related to its better cost effectiveness.

### (4) Convenience

Statements stating the comfort of patient, e.g., improved dose, low frequency of dosage, ease of administration, etc. with or without reasons to support the same.

### (5) Pharmacokinetic property

Properties of the drug related to its absorption, metabolism, half-life, etc.

### (6) Pharmaceutical property

New dosage formulations, different manufacturing procedures, excepients (e.g., cyclodextrin, sodium bicarbonate, etc.), storage facilities, etc.

### (7) Extravagant emotional claims

Any claims or statements not related to patient outcome, disease outcome, or drug, e.g. 1^st^ of its kind in the world or India, flavored oral preparations, authentic certification of manufacturing plant (GMP certification, USFDA approval, etc.), various drug formulations, packaging characteristics, or technologies of the drug production. Those were promoted using non-universal abbreviations and pharmaceutical jargons, e.g. FFS pack, HHT pack, Alu-Alu pack or drug impregnated pellet system (DIPS), multiple unit pellet system (MUPS), Eudragit coating (flash tab system), Glatt coat, RTT technology, bilayered technology, international immediate release bicarbonate technology, etc.

Promotional literature quoting references in support of the claims made in it was further evaluated for its authenticity. References sustaining the claims were categorized as per the source of material, i.e. journals, web sites, books, data on file and other including guidelines, seminar, departmental studies, and prescription information.

Further Internet search was done to retrieve the references mentioned in the brochures. We considered a reference as “available” if we could obtain a softcopy of the cited material in the National Library of Medicine’s PubMed or website of mentioned journal, freely in either full text or abstract format. References other than journal articles were searched through Google meta search engine. As a corollary data on file, departmental studies and references not available from the search were considered nonretrievable. The available journal references were grouped as per the type of study quoted in it or the type of article as follows:

### Research article describing

- Randomized controlled trial (RCT)
- Observational studies (prospective or retrospective noninterventional studies, Pharmacovigilance studies, case–control studies)
- Clinical trials (without specific details of design)
- Review article
- Meta-analysis
- Preclinical studies (*In vitro* studies and animal studies)
- Others (Editorial, Letter to editor, Correspondence, etc.)

The available references were checked with the claims made in the promotional brochures. Depending upon their presentation, we classified them as either validly, invalidly, or partially validly presented. Validity assessment of the references and type of reference were independent, i.e. if referenced article was quoting a RCT but if its outcome was manipulated while supporting the claim, it was considered invalid reference and *vice versa*.

Promotional materials are usually made attractive using various pictures or various methods of data presentation to persuade doctors for prescribing the drug promoted in it. Pictorial content of the promotional brochures was evaluated for:

1. The type of pictures (healthy babies, women, patients, doctors, medicinal products, or other treatment unrelated pictures) and
2. Number of scientific tables,
3. Scientific graphs, and

Pseudographs—psuedograph is a graphical presentation without proper axes, labeling legend, or just arrows with numbering showing reduction or increase.[17]

Promotional brochures giving brief prescription information (BPI) were assessed to calculate percentage of total area used to give this important information. Area was calculated by measuring breadth and width of space occupied by BPI using transparent ruler in cm^2^. Similarly, the total area of literature was calculated to find how much percentage of it was utilized for BPI.

## Results

### (1) Type of drug

**Figure 1:**
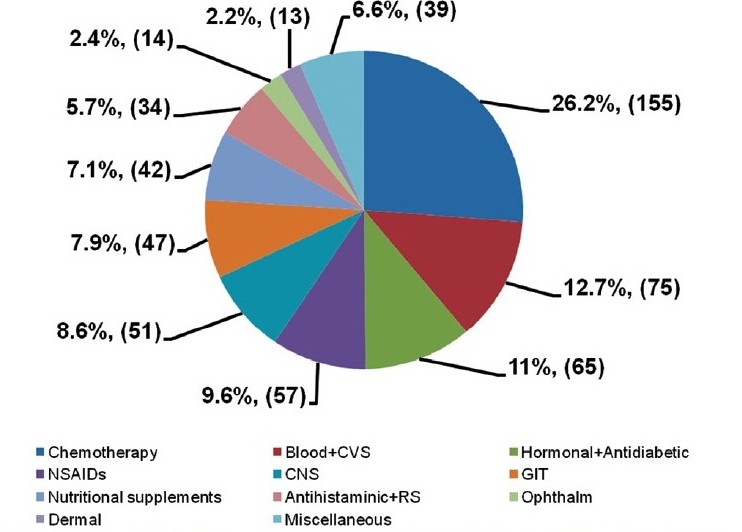
Classification as per type of drug promoted in the literature (n = 592). Miscellaneous→ Vaccines and sera, analgesic, anti-inflammatory, antispasmodic, antiseptic, anticholinergic and muscle relaxant

A total of 592 drugs were presented in 513 drug promotional brochures. Out of 592 promoted drugs, 41.1% were fixed dose combinations (FDCs) whereas 58.9% were single drug preparations. Out of 243 promoted FDCs, only 35 (14.4%) were recommended by WHO [Figure 1].

Chemotherapeutic agents were the most promoted group (26.2%), and they can contribute to development of drug resistance, due to overuse. Cardiovascular drugs (12.7%) and hormones (11%) come close second and third among promoted drugs. These are the medicines once started, are taken for lifelong, providing long-term financial benefit to the pharmaceutical companies.

### (2) Fulfillment of WHO criteria

Our findings showed that pharmaceutical industries were most reluctant to provide information regarding treatment regimen, information regarding safety and adjuvant, rather promotion was only focused on latest drug formulations and these were supporting the findings of previous studies.[1,18,19] It was found that none of the brochures fulfilled all the 10 WHO criteria. After excluding lowest presented criteria, i.e. adjuvant, rest nine criteria were fulfilled by only 6.1% literature. Approximately half (48.7%) of the evaluated brochures were satisfying only six criteria, namely brand name, INN, dosage form, approved use/s, content of the active ingredients, and address of the manufacturer [Table 1].

**Table 1:** Evaluation of promotional literature as per WHO criteria (n = 513)

*Criteria*	*Mentioned number (%)*
INN^a^	492 (95.9)
Brand name	513 (100)
Content^b^	408 (79.5)
Adjuvant	10 (1.9)
Approved therapeutic use/s^c^	443 (86.3)
Dosage form	447 (87.1)
Regimen^d^	165 (32.2)
Safety information	45 (8.8)
Manufacturer address	362 (70.6)
References to scientific information	316 (61.6)

a International nonproprietary name,

b Active drug per dosage form,

c Drug use approved as per Central Drugs Standard Control Organization (CDSCO),

d Drug dose, frequency, and duration of drug

### (3) Claims

Apart from giving therapeutic information, pharmaceutical companies also made multiple claims, as much as six per literature. Brochures making two claims each were the maximum in number 31.2%. A total of 1171 claims were made in promotional brochures. Claims about efficacy were made in 92% brochures, followed by that of safety in 37.8% and of pharmaceutical properties in 29.6%.

Number of promotional brochures making extravagant emotional claims, claims regarding cost effectiveness, pharmacokinetic properties, and convenience were almost equal in number, i.e. 17.9% (92), 17.7% (91), 16.7% (86), and 16.4% (84), respectively. Our findings were similar to the observations made in other studies where author found broad unscientific, dubious claims in promotional literature.[4,15]

### (4) References

**Figure 2:**
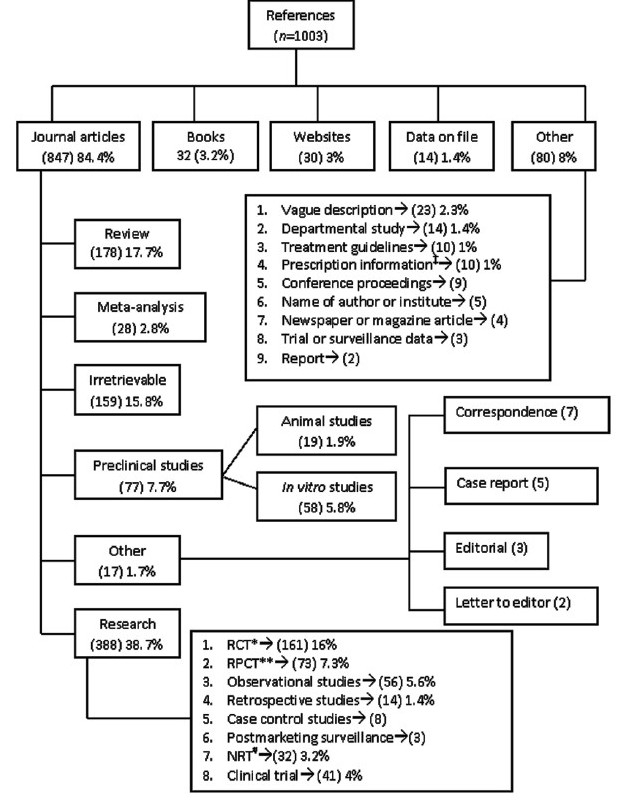
Classification of references given in the drug promotional literature ‡Prescription information includes brief prescription information from pharmaceutical company’s web site (7), physicians’ desk reference (2) and ‘pharma aid’ (1); *lRCT→ Randomized controlled trial; **RPCT→ Randomized placebo controlled trial; #NRT→ Nonrandomized clinical trial

Pharmaceutical industries gave references to support information presented or claims made in the drug promotional brochures. One-third of promotional brochures 33.3% cited one or two references. References in the range of three to seven per brochures were given by 24.2%. Pharmaceutical industries’ behavior was very surprising that on one hand eight to nineteen references per brochure were given in 4.1% brochures and on other side 38.4% of it did not bother to give any to support the claims made in it. Classification of references distinctly demonstrates that citations from journal articles were the maximum (84.4%) [Figure 2].

### (5) Retrievability and validity of references

The maximum retrieved references were the journal article abstracts, from which the related details were identified and then classified. Major share of journal articles was contributed by research articles and review articles. Half of the given references (49.3%) were presented validly and research articles (28.5%) had a major quarter. Rest half of the references were either invalidly presented 22.3%, irretrievable 21.9%, or partially valid in presentation 6.4%. References giving the results of clinical trials of ambiguous methodology or nonrandomized clinical trials were 7.3%. These findings were similar to those of Villanueva *et al*., van Winkelen *et al*. and Cooper and Schriger.[5,20,21] All the animal study references (1.8%) except for one were invalid. Claims outnumbered the references provided in the promotional literature, and 440 claims from 197 promotional brochures were without any reference. In the absence of one to one correspondence in the claims and references, we specifically checked all safety claims, as per WHO criteria for their substantiation. Although WHO criteria strictly states that ‘the word “safe” should be used if properly qualified,’[16] findings of our study were presenting the facts proving irresponsible behavior of pharmaceutical industries. Out of 194 leave behinds claiming for safety of drug presented in it, only 27.8% gave supporting reference while 72.2% boosted the same without any reference. Current finding underlines the profit driven promotional attitude of the pharmaceutical industries, which is depriving the physicians of authentic drug information [Table 2].

**Table 2:** Classification of references as per validity of its presentation (n = 1003)

*Type of reference*	*Type of presentation*				*Total*
	*Valid no (%)*	*Partial no (%)*	*Invalid no (%)*	*Not retrievable no (%)*	
Research article#	286 (28.5)	31 (3.1)	71 (7.1)	–	388
Review article	121 (12.1)	19 (1.9)	38 (3.8)	–	178
Meta-analysis	17 (1.7)	7	4	–	28
*In vitro* study	32 (3.2)	6	20 (2)	–	58
Animal study	1	0	18 (1.8)	–	19
Book	7	0	1	24 (2.4)	32
Website	8	0	9	13 (1.3)	30
Data on file	0	0	0	14 (1.4)	14
Other journal article^a^	14 (1.4)	0	3	–	17
Other references^b^	9	1	33 (3.3)	37(3.7)	80
Journal article not retrievable^c^	–	–	–	132(13.2)	159*
Journal citation^d^	–	–	27 (2.7)	–	
Total	495 (49.3)	64 (6.4)	224 (22.3)	220 (21.9)	1003

# Research articles include randomized controlled trials, randomized placebo controlled trials, nonrandomized trials, clinical trials without details of design, observational studies without details of design, retrospective studies, case–control studies, postmarketing surveillance studies;

a Other journal article includes Case report, Correspondence article, Editorial, and Letter to editor;

b Other references include conference proceedings, report, departmental study, therapeutic guidelines, name of author, name of institute newspaper article, health magazine article, unpublished trial or surveillance data, online medicine prescription information, physicians’ desk reference (PDR), “pharma aid,” and reference with vague description;

c Article not retrievable using reference provided in the corresponding promotional brochure

* Sum of “c” and “d.”

d Journal citations invalid because of typographical error;

### (6) Pictures

**Figure 3:**
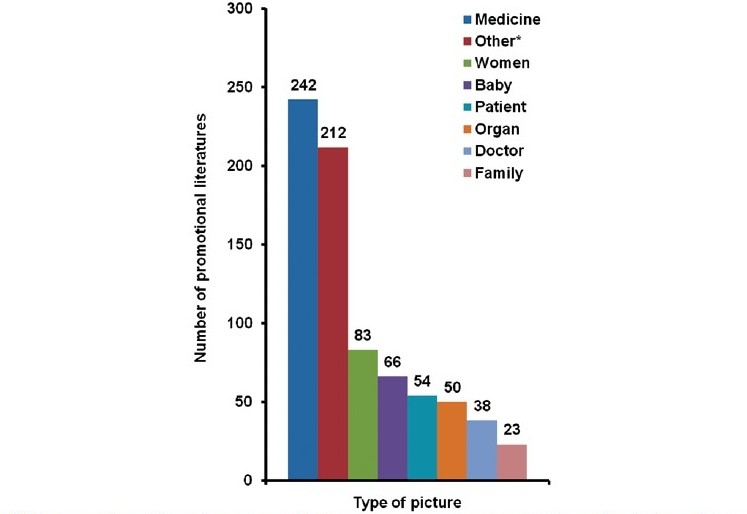
Categorization of pictorial content in the drug promotional literature (n = 768)# #463 promotional literature presented one or more than one picture; *includes picture of cars, cricket, scenery, horoscope information, cartoon, artistic figure, etc.

As a part of persuasive communication, these promotional brochures were made striking using various types of pictures and devoting major of the literature area to nonspecific content depriving it of useful therapeutic information. It was type of picture and not the number of pictures per brochure, which we counted while evaluating the literature. Our observation that 90.2% brochures contained such irrelevant pictures supported the studies done by Stimson and Cooper *et al*.[1,17] Study by Curry and O’Brien has shown that “male heart patients” and “depressed female patients” were stereotyped through the images used in the advertisements, which could strengthen gender association.[22] We were unable to find such an association though pictures of women was forming third large category in varied types of pictures presented in the evaluated brochures [Figure 3].

Brochures presenting pictures unrelated to medicine, disease, or therapy were 41.3% representing the tendency of pharmaceutical industry of wasting money in printing eye catching glossy paper promotional brochures deprived of important therapeutic information. This fact is proved by the information that BPI of the promoted drug was given only by 8.8% brochures. This finding was similar to that of study by Lohiya.[23] Also, 60% of it spared just less than 10% of total area with the mean of 4.08% for presenting this important information. Use of such a negligible area for BPI made this information poorly legible on the contrary brand names and claims were displayed in the bold so as to make an impact.

We found that some of the evaluated drug promotional brochures used graphical presentations to depict the information. A total of 219 graphical presentations were evaluated from 15.9% of drug brochures. Pseudographs (28.8%) were the maximum in number followed by tables (25.6%) and bar diagrams (23.3%). Line diagrams (10.9%), tables giving cost comparisons (7.3%), pie diagrams (3.6%), and scatter diagram embraced the rest of graphical presentations. The predominance of pseudographs pointed out the commercial and promotional rather than educational attitude of the industries. Our findings were consistent with those of the study by Cooper *et al*.[17]

## Discussion

It was concluded from this study that pharmaceutical industries did not follow WHO guidelines while promoting their drug products, thus accelerated their commercial motive rather than ethical educational aspect. Little therapeutic information was provided to help physicians reach any rational decision about promoted drug. Promotional activity was concentrated not much on innovative medicines’ exposure, but on publicizing fixed dose combinations, not recommended by WHO. The promotional brochures were full of unsubstantiated claims regarding safety or efficacy, and those claims were therapeutically irrelevant also. Important information regarding adverse drug reactions, contraindications, or drug interactions was usually missing. Reference citations were given to earn credibility, but it was difficult to trust them because of ambiguous presentation, poor quality, and questionable retrievability. Therapeutically unrelated matter was printed, compromising the space to be given to important BPI.

Printed promotional material is an important source of information. On the basis of the observations of this study, it is suggested that physicians need to be aware of the flaws in promotional literature before accepting it as valid information. This could help monitor it with great vigilance. The association of pharmaceutical companies in developed countries, e.g. UK, Australia, and Canada are required to observe a code of practice in marketing as a signatory condition for membership of the association.[24] India has set up regional ethics committee to collect complaints against unethical drug promotion advertisements at Mumbai, New Delhi, Chennai, and Chandigarh which forward these complaints to drug controller authority to take necessary legal steps to discipline guilty companies.[11,25] Forwarding more complaints about irrational promotion to regulatory authority by cautious doctors might lead pharmaceutical industry to incline toward self-regulation. Government regulatory bodies must play a proactive role where code of ethics is failing. Wherever the hospitals are attached to the academicia, prior scrutiny of the promotional material for authenticity of the content could be done by respective department of pharmacology.

This study evaluates one type of promotional activity of pharmaceutical company, i.e. printed promotional literature; however, interventional research to assess the awareness of the physicians about these facts and alerting them about the same will help gain accurate and ethical information from promotional literature.
